# Clinical outcome of 23g Trans-Conjunctival pars plana vitrectomy - a prospective comparison of Phaco-Vitrectomy with only vitrectomy in phakic eyes

**DOI:** 10.12669/pjms.335.13430

**Published:** 2017

**Authors:** Haroon Tayyab, Asad Aslam Khan, Rana Muhammad Mohsin Javaid

**Affiliations:** 1 Dr. Haroon Tayyab, FCPS (Ophth), FCPS (Vitreoretinal Ophthalmology), FICO, Department of Ophthalmology, King Edwards Medical University - Mayo Hospital, Lahore, Pakistan; 2 Prof. Dr. Asad Aslam Khan, (SI) - MS (Ophth), PhD, Department of Ophthalmology, King Edwards Medical University - Mayo Hospital, Lahore, Pakistan; 3 Dr. Rana Muhammad Mohsin Javaid, FCPS, Department of Ophthalmology, King Edwards Medical University - Mayo Hospital, Lahore, Pakistan

**Keywords:** Macular hole, Pars plana vitrectomy, Phacoemulsification, Retinal detachment, Vitreous haemorrhage

## Abstract

**Objective:**

To evaluate the effectiveness and safety profile of combined phacoemulsification with 23G pars plana vitrectomy when compared to pars plana vitrectomy alone in phakic patients.

**Methods:**

This study was performed at Al-Ehsan Eye Hospital (tertiary care eye hospital in Lahore, Pakistan) from January 2016 to August 2016. A total of 40 eyes in two equal groups of 20 eyes each, were enrolled in this prospective study. Group-A underwent combined phaco-vitrectomy, whereas Group-B underwent vitrectomy only for various vitreoretinal pathologies. We evaluated the safety of combined surgery, intra-operative and postoperative complications and short term surgical outcome.

**Results:**

The most common reason for vitreoretinal intervention was rhegmatogenous retinal detachment followed by vitreous haemorrhage in combined study population. There was statistically significant difference in best corrected visual acuity pre-operatively and post operatively within the groups and between the groups. The most significant immediate post operative observation in Group-A was enhanced anterior chamber inflammation as compared to Group-B, whereas most signification observation in Group-B was development of visually significant cataract (35%) at 6 months follow-up. There was no other significant sequel or complication difference between both groups.

**Conclusions:**

Combined phaco-vitrectomy is a safe and effective procedure with minimum complication profile and it avoids the need of subsequent cataract surgery.

## INTRODUCTION

With the recent advances in small gauge cataract surgery, the threshold of combining phacoemulsification with other intra and extra ocular surgeries has come down.^1^ With this growing ease in handling complex surgeries, many vitreoretinal surgeons are preferring to perform pars plana vitrectomy (PPV) with phacoemulsification in a single sitting.^2^ Although merits and demerits of combined procedures will be discussed here, it is not always the surgeons choice to perform both procedures together. Various vitreoretinal facilities across Pakistan may have differing protocols depending upon the logistics and expertise of the surgeons. Most upcoming and practicing vitreoretinal surgeons in Pakistan are well trained in phacoemulsification due to nature of the training programs where vitreoretinal surgery is taken as second speciality rather than primary speciality unlike centres in USA. Combined surgical procedures have a significant learning curve.

Ever since the introduction of small gauge transconjunctival PPV, the availability of various treatment options has remarkably increased for ophthalmologists and patients alike.^3^,^4^

Combined phacoemulsification and PPV essentially eliminates the need of second surgical procedure (cataract extraction) and provides better long term visual results. Also, when sequential phacoemulsification is performed, the rate of complications in phacoemulsification surgery is higher due to difficulty of surgery in vitrectomized / silicone oil filled eye and the formation of hard cataract in vitrectomized eye.^5^

There are many reports of combined surgical procedures and surgeons have employed various techniques to achieve desirable results.^6^,^7^ In this study we have reported our experience of combining phacoemulsification with PPV as a single sitting procedure and compared its results and complications with PPV alone in phakic eyes.

## METHODS

This prospective non-randomised study was performed at Al-Ehsan Eye Hospital (tertiary care eye hospital in Lahore, Pakistan) from January 2016 to August 2016. All patients gave consent for phacoemulsification with intraocular lens (IOL) implant along with already discussed PPV for their retinal pathology. This study was approved by hospital ethics committee. All surgeries were performed by a single vitreoretinal surgeon. A total of 40 eyes of 40 patients were included in this study. Twenty eyes were in Group-A (combined phacoemulsification and IOL implant with PPV) whereas 20 patients were in Group-B (PPV only). All patients were followed up for six months post surgery.

Patients who presented with dropped nucleus, endophthalmitis and penetrating injury were not included in this study. All patients in Group-A had some degree of cataract in their eyes and all patients in Group-B had clear crystalline lens.

We used Faros vitrectomy machine (Oertli Instrumente AG, Switzerland) for performing anterior and posterior segment surgery. For retinal visualization, we used Oculus BIOM 2 with Oculus SDI Inverter 2 (OCULUS Surgical, Inc. Port St. Lucie, USA). All patients underwent 23G transconjunctival PPV for various retinal pathologies. Other adjuncts used in these surgeries included GOT Multi SF6 - pure sulphur hexafluoride, GOT Multi C3F8 - pure octafluoropropane gas, RS-OIL ECS Silicone oil 1.000 cS, TWIN 018 HD 0.18% trypan blue + 0.03% blulife, HPF10 high purity perfluorodecalin (AL.CHI.MI.A. SRL - - Viale Austria). We used Vitra Multisopt 532nm green laser (Quantel Medical, Bozeman - US) for endolaser.

In all cases of Group-A, three port 23G self sealing cannulas were inserted at 3.5mm from limbus at standard quadrants before starting phacoemulsification. All phaco incisions were near clear corneal and 2.8mm in length. Routine phacoemulsification was performed and IOL was implanted at the end of PPV. We used acrylic hydrophilic square edge foldable IOLs. Corneal incisions were secured with 10 0 Nylon suture at the end of cortical cleanup. All vitrectomy ports were closed with 7 0 Vicryl suture.

We used SPSS statistical software (version 20.0; SPSS Inc., Chicago, Illinois, USA) for data analysis. Numerical data was represented as mean +/- standard deviation. For the rest we used percentages. We used Chi-square test as test of significance and P<0.05 was considered statistically significant.

## RESULTS

**Fig.1:**
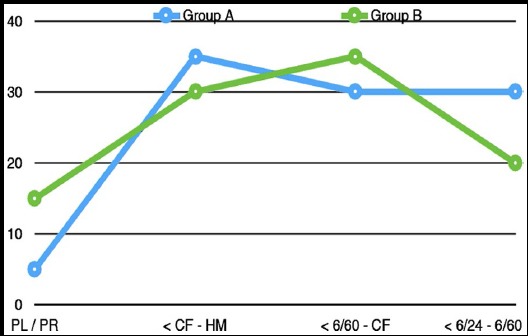
Pre-operative best corrected visual acuity range for Group-A and B.

A total of 40 eyes of 40 patients were included in this study. Twenty eyes were in Group-A (phaco-vitrectomy) and 20 eyes were in Group-B (PPV only). There was no significant difference in the mean age of patients in both groups (53.1±9.61 years in Group-A and 51.6±10.05 years in Group-B; p-value >0.05). In Group-A, 11/20 patients were male whereas in Group-B 9/20 patients were male. The most common reason for vitreoretinal surgery in Group A and B are listed in Table-I. Pre-operative best corrected visual acuity range for Group-A and B is shown in the following Fig.1. We used C3F8 intra-ocular gas in 14 (35%) patients, silicone oil (1000cS) in 12 (30%), SF6 in 6 (15%), air in 3 (7.5%) and basic salt solution (BSS) in 5 (10%) patients.

**Table-I:** Shows most common reason for vitreoretinal surgery in Group A and B.

*Retinal Pathology*	*Group-A n (%)*	*Group-B n (%)*
Rhgmatogenous Retinal Detachment	6 (30%)	8 (40%)
Vitreous Haemorrhage (multiple aetiology)	6 (30%)	5 (25%)
Diabetic Tractional Retinal Detachment	3 (15%)	5 (25%)
Epiretinal membrane (multiple aetiology)	3 (15%)	1 (5%)
Vitreomacular Traction	1 (5%)	1 (5%)
Idiopathic Macular Hole	1 (5%)	0 (0%)

**Fig.2:**
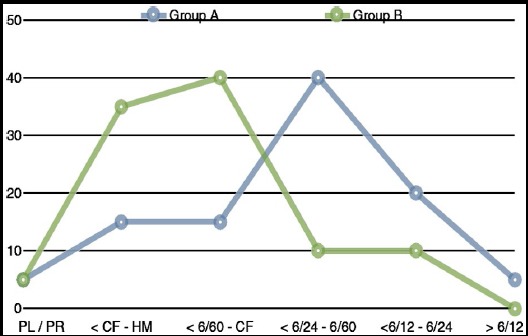
Best corrected visual acuity at 6 months follow-up for Group A and B.

First week post-operative BCVA was significantly lower in patients receiving gas and air tamponade as compared to patients having BSS in the vitreous cavity. This statistically significant difference (p-value < 0.05) in first week post operative BCVA was attributed to the inherent properties of gas and air in vitreous cavity to impair vision. Patients with silicone oil had intermediate decrease in BCVA. This difference in first week BCVA was also statistically significant within the groups and between the group’s pre and post operatively. The BCVA at 6 months follow-up is shown in Fig.2.

During the study period, 7 (35%) eyes in Group-B developed visually significant cataract and 2 (10%) eyes in Group-A developed visually significant posterior capsular opacity. There were no incidents of posterior capsular rupture (PCR). There were no reports of IOL capture or IOL decentration in Group-A. However, on 1st day follow-up, Group-A had 4 (20%) patients with AC fibrin reaction compared to only 1 (5%) patient in Group-B. Three out of these 4 patients in Group-A were diabetics. This difference was statistically significant (p < 0.05). Two patients (10%) in Group-A had posterior synechie (PS) at end of 1st week follow-up and one of these patients had inadequate pupil dilation because of this problem. No such event happened in Group-B and difference was significant (p-value <0.05). There was one report of crystalline lens touch by the surgeon in Group-B who developed accelerated cataract after PPV and needed early phacoemulsification with IOL implant. There were two cases in each group who had corneal oedema at first post operative day that settled with topical medications. The difference was statistically insignificant (p-value > 0.05). There were no other untoward complications in the immediate post operative period.

Intraocular pressure (IOP) was similar in both groups preoperatively (14.4±3.1 mm Hg in Group-A and 14.35±3.21 mm Hg in Group-B; p-value > 0.05). The difference was statistically insignificant. However, on 1st post operative day, mean IOP in Group-A was higher than in Group-B (17.1±4.03 mm Hg vs 13.6±3.42 mm Hg, respectively, p-value < 0.05). One eye in Group-A had persistent IOP of 28 mmHg due to overfilled silicone oil. We had to remove some silicone oil at end of two weeks to restore normal IOP. At 6 months follow-up, there was no significant difference in mean IOP of both groups.

## DISCUSSION

As the surgeons are becoming more comfortable with small gauge PPV and with the advancements in phacoemulsification with small incisions, there is an increasing trend towards combining both the procedures in single sitting.^8^ This combined surgery has been performed and reported for various vitreoretinal pathologies like rhegmatogenous retinal detachment, advanced diabetic eye disease, macular holes, vitreomacular interface disorders etc with good success rates and minimal complication profile.^9^ Most common sequel of an eventless PPV in phakic eyes is accelerated development of nuclear sclerosis.^10^ Also, it’s common observation that cataract surgery in vitrectomized eye is more challenging than routine cases due to harder nucleus, variable and unstable AC dynamics, weak zonules and possibility of posterior capsular damage during PPV. Also the fact that PPV itself is becoming smaller gauge, sutureless and with faster rehabilitation; and the combined procedure being considered safe and effective has resulted in shift towards phaco-vitrectomy done in single sitting.^10^,^11^

There has been slight variation in the technique involved in performing combined procedure. Some surgeons have used 7 mm optic intraocular lens to avoid the edge effect of IOL during peripheral vitrectomy like vitreous base shave. Others have implanted IOL before performing PPV while not reporting any edge effect of IOL (6 mm optic).^10^ We preferred to implant IOL at the end of PPV and do not report any significant difficulty during PPV. We always applied a single 10/0 nylon suture to all corneal wounds after phacoemulsification that was removed at the end of surgery. While reviewing the literature, we found that surgeons performing Micro Incisional Cataract Surgery (MICS) did not apply corneal suture due to small incision size (1.8mm compared to 2.7mm).

The most important intraoperative complication dreaded during phacoemulsification is PCR. We did not report any PCR in our study whereas the rate for PCR in other similar studies has been between 3 - 4.2%.^10^,^12^ In our study, we did not encounter any IOL capture or IOL decentration. We placed special emphasis on a very controlled capsulorrhexis and were able to maintain the size of capsulorrhexis to nearly 5.5mm and central in location. In contrast, there have been reports of IOL tilt and IOL capture. Our results were similar to Arikan et al. who also reported no immediate post operative IOL capture whereas *Demetriades reported* cases with IOL capture and tilting. He further emphasized that the diameter of capsulrrhexis was not controlled in his patients.^9^,^13^

Anterior Chamaber inflammation and fibrin deposition is another concern with combined phaco-vitrectomy. We reported 4 (20%) patients in whom there were grade 4 anterior chamber reactions with fibrin deposition. Our reported rate is higher than comparative studies who reported such occurrence limited to 3.5 - 7%.^7^ One reason was that three out of these four patients were diabetics who had undergone diabetic vitrectomy for vitreous haemorrhage and TRD. Another reason could have been the use of non branded ophthalmic viscoelastic device (OVD) and probable incomplete washout of OVD from AC at the end of surgery. Tosi et al. recently reported day one fibrin deposition in AC to be like our study (6 out of 30 patients; 20%).^14^ He did not comment reason of such findings in his study. Lower rates of AC fibrin deposition and synechie formation have also been reported by other investigators.^15^-^18^ We reported statistically significant (p-value < 0.05) difference in AC reaction and fibrin deposition in both the groups. Two patients in Group-A had posterior synechie of whom one successfully responded to topical mydiatrics and resolved by the end of study period.

It has been proposed that inflammation, endotamponade and posturing contribute to earlier PCO formation in phaco-vitrectomy patients.^10^ We reported a 10% occurrence of PCO formation in patients who had undergone combined phaco-vitrectomy. But, this reported rate can be misleading and should not be generalized because of our shorter 6 month follow-up period and use of hydrophilic IOL. Other studies have reported the incidence of PCO formation ranging from 5.8% to 51.1% with variable follow-up periods.^10^,^19^-^21^

## CONCLUSION

This study has demonstrated that combined PPV and phacoemulsification is a safe and effective procedure in comparison with PPV alone in phakic patients. Also this combined procedure obviates the need of second surgery of cataract extraction later on. Good points in this study include its prospective case control design and single surgeon. The limitations include small study population, relatively brief follow-up period and multitude of retinal pathologies.

### Authors’ Contributions

**HT** was the primary surgeon, did review of literature and data collection.

**ASK** conceptualized the study and did manuscript final editing.

**RMM** did data analysis and manuscript writing.
